# 

**Published:** 1879-11

**Authors:** 


WHOLE NUMBER, CCCCXIV.

**November, 1879.**

NUMBER 5, VOL. XXXIX.

THE

### CHICAGO MEDICAL Journal and Examiner

INDEX MEDICUS.

S.G.O. INDEXED

**EDITORS:**

WILLIAM H. BYFORD, A.M., M.D.,

JAS. NEVINS HYDE, A.M., M.D. N. S. DAVIS, M.D., LL.D.

DANIEL R. BROWER, M.D.

CHICAGO:

*PUBLISHED BY THE CHICAGO MEDICAL PRESS ASSOCIATION.*

188 CLARK STREET.

☞ Read the Notice of this Journal among Advertisements.

